# Distinct phosphorylation signals drive acceptor versus free ubiquitin chain targeting by parkin

**DOI:** 10.1042/BCJ20210741

**Published:** 2022-03-28

**Authors:** Karen M. Dunkerley, Anne C. Rintala-Dempsey, Giulia Salzano, Roya Tadayon, Dania Hadi, Kathryn R. Barber, Helen Walden, Gary S. Shaw

**Affiliations:** 1 Department of Biochemistry, The University of Western Ontario, London, Ontario N6A 5C1, Canada; 2 Institute of Molecular Cellular and Systems Biology, University of Glasgow, Glasgow G12 8QQ, U.K.

**Keywords:** fluorescence, phosphorylation, protein structure, protein–protein interactions, ubiquitin, ubiquitin ligases

## Abstract

The RBR E3 ligase parkin is recruited to the outer mitochondrial membrane (OMM) during oxidative stress where it becomes activated and ubiquitinates numerous proteins. Parkin activation involves binding of a phosphorylated ubiquitin (pUb), followed by phosphorylation of the Ubl domain in parkin, both mediated by the OMM kinase, PINK1. How an OMM protein is selected for ubiquitination is unclear. Parkin targeted OMM proteins have little structural or sequence similarity, with the commonality between substrates being proximity to the OMM. Here, we used chimeric proteins, tagged with ubiquitin (Ub), to evaluate parkin ubiquitination of mitochondrial acceptor proteins pre-ligated to Ub. We find that pUb tethered to the mitochondrial target proteins, Miro1 or CISD1, is necessary for parkin recruitment and essential for target protein ubiquitination. Surprisingly, phosphorylation of parkin is not necessary for the ubiquitination of either Miro1 or CISD1. Thus, parkin lacking its Ubl domain efficiently ubiquitinates a substrate tethered to pUb. Instead, phosphorylated parkin appears to stimulate free Ub chain formation. We also demonstrate that parkin ubiquitination of pUb-tethered substrates occurs on the substrate, rather than the pUb modification. We propose divergent parkin mechanisms whereby parkin-mediated ubiquitination of acceptor proteins is driven by binding to pre-existing pUb on the OMM protein and subsequent parkin phosphorylation triggers free Ub chain formation. This finding accounts for the broad spectrum of OMM proteins ubiquitinated by parkin and has implications on target design for therapeutics.

## Introduction

Parkinson's disease (PD) affects ∼1% of the world's population [1]. Though age of onset and disease severity can vary greatly among patients, there is increasing evidence that implicates mitochondrial dysfunction as a major contributor to neurodegeneration [2]. A number of genes have been associated with familial PD but mutations in two genes in particular are found in over 50% of patients: *PARK2*, which encodes the ‘RBR’ E3 ubiquitin ligase parkin, and *PARK6*, which encodes PTEN-induced kinase 1 (PINK1) [3]. Under oxidative stress conditions PINK1 activates parkin (pParkin), promoting the ubiquitination of a wide array of proteins on the outer mitochondrial membrane (OMM) as a signalling step for mitophagy [4–6].

Parkin is an intricate E3 ligase that possesses an N-terminal ubiquitin-like (Ubl) domain and C-terminal RING0, RING1, in-Between-RING (IBR) and RING2(Rcat) domains (R0RBR). Ubiquitination by parkin requires recruitment of an E2∼ubiquitin (E2∼Ub) conjugate and transfer of ubiquitin (Ub) from the E2 (UBE2L3) to a catalytic C431 in the RING2(Rcat) domain prior to substrate labelling. Under basal conditions parkin is localized to the cytosol and displays minimal ubiquitination activity. This is a result of auto-inhibition by the N-terminal Ubl domain and a unique Zn^2+^-binding RING0 domain, which block E2∼Ub recruitment by the RING1 domain and access to the catalytic cysteine in the RING2(Rcat) domain, respectively [7–12].

Oxidative stress models induced by mitochondrial oxidative phosphorylation uncouplers (FCCP, CCCP, oligomycin/antimycin) are used in cells to study parkin-mediated ubiquitination by stimulating reactive oxygen species production [13]. This stabilizes PINK1 at the outer mitochondrial membrane (OMM) resulting in both recruitment and increased ubiquitination activity of the E3 ligase parkin. Translocation of parkin to the OMM is controlled by PINK1 phosphorylation at S65 in ubiquitin (pUb) within existing K48- and K63-linked ubiquitin chains [14,15]. Structural studies show pUb binds to a cleft between the RING0 and RING1 domains, modifies the arrangement of the IBR domain and weakens the association of the Ubl domain in parkin [11,12,14,16,17]. Multiple *in vivo* and *in vitro* studies show that pUb, or phosphorylated ubiquitin chains, trigger increased ubiquitination by parkin and result in accelerated unloading of an E2∼Ub conjugate [11,12,14,16].

Ubiquitination activity by parkin is also controlled by PINK1 phosphorylation of S65 within its Ubl (pUbl) domain [18–20]. This step requires prior recruitment by pUb since parkin phosphorylation is negligible in cells due to lack of translocation to the OMM [21–23]. Structurally, in the absence of pUb binding, the S65 phosphorylation site in the Ubl domain is protected by residues in the tether region of parkin [24]. In the presence of pUb, the position of the Ubl domain is modified [11,12,24] and once phosphorylated, pUbl has the potential to relocate to a basic patch (K161, K211) in the RING0 domain [25,26] although this is not evident in a structure of phosphomimetic (S65D) parkin bound to pUb [27]. The pParkin:pUb complex is frequently referred to as the ‘fully activated’ state that promotes transfer of Ub from an E2∼Ub conjugate to C431 in the RING2(Rcat) domain needed for ubiquitination of OMM proteins [15,16,22,25,26,28]. *In vitro* and *in vivo* experiments show that phosphorylation of both ubiquitin and parkin leads to increased activity compared with pUb alone. However, deciphering the distinct steps in this mechanism is difficult due to the complexity of ubiquitination products formed, frequently including parkin autoubiquitination, free Ub chain formation, and ubiquitination of substrates [11,27,29,30]. Furthermore, of the many substrates suggested for parkin, most experiments result in only mono- or di-ubiquitinated products and rarely show the extensive ubiquitination patterns that are observed for autoubiquitination of parkin or free ubiquitin chains [16,22,27,30–32].

Cellular studies also show that different substitutions in parkin alter translocation to the mitochondria under oxidative stress conditions, including substitutions at the Ubl phosphorylation site (S65) and in the RING0 domain (K161, K211). Some of these substitutions show opposing effects in the rates or degree of translocation depending on cell type or technique [33–36]. Not surprisingly, total ubiquitination decreases as pUb-dependent translocation of parkin is impaired. However, some *in vivo* studies show parkin that is unable to be phosphorylated (S65A) retains the ability to ubiquitinate OMM proteins while others report a lack of activity [33–35,37]. In addition, in the presence of pUb, S65A-parkin discharges an E2∼Ub conjugate and forms a parkin∼Ub conjugate similar to wild-type parkin [27]. The ubiquitination of OMM substrates is further complicated because immunoprecipitation assays show minimal interaction between parkin and some substrates including Miro1 [35,38] and mitofusin 2 [39], though it is enhanced following pUb production from oxidative stress. Furthermore, inspection of OMM substrate sequence information does not reveal a common parkin recognition sequence, motif or predictable ubiquitination site. Despite the many strengths of *in vivo* experiments, these observations have made it difficult to elucidate and quantify certain mechanistic details regarding OMM substrate ubiquitination.

In this work we aim to distinguish the mechanisms used by parkin to select OMM proteins for ubiquitination as compared with autoubiquitination and free Ub chain formation. Since PINK1 phosphorylation at S65 of ubiquitin in polyubiquitin chains has been established as an essential step for parkin activity *in vivo* [14,15,23], we created a series of unphosphorylated and phosphorylated substrate-Ub chimeric proteins, or Ub ‘acceptors’, to examine their direct recruitment and strengths of interaction with parkin. Furthermore, we have used fluorescent tags that allowed us to quantify the ubiquitination of an OMM protein, parkin autoubiquitination or free Ub chain synthesis. Surprisingly, we find that parkin prefers to bind to a pre-attached pUb molecule, ligated to an OMM protein (acceptor), and not the OMM protein itself. Rather than phosphorylation of parkin, this recognition of the pUb molecule is essential for ubiquitination of the acceptor protein. In contrast, phosphorylation of parkin moderately enhances acceptor-pUb protein ubiquitination but greatly stimulates free Ub chain formation. These observations provide a new rationale for the tight interdependence of pUb translocation and ubiquitination activity for parkin.

## Materials and methods

### Molecular biology

DNA for the GTPase2 domain of human Miro1 (mG2, residues 402–582) and human CISD1 (residues 32–108) were codon optimized and inserted into His_6_- expression plasmids (ATUM, Newark, CA, U.S.A.). The mG2 protein contained 3 substitutions (V418R, Y470S, L472A) shown to stabilize the monomer form of the protein [29]. For some experiments a fourth substitution (I459T) was introduced to improve solubility. For solubilization of mG2, an smt3 tag was inserted after the His_6_-tag by Restriction-Free (RF) cloning [40,41]. For constructs that required fluorophore labelling using DyLight 800 or Alexa Fluor 680 (see below), a single Cys residue was inserted N-terminal to the protein coding sequence by site-directed mutagenesis.

The chimeric ‘acceptor-Ub’ species were created by inserting the yeast Ub sequence C-terminal to the substrate, including a Ser-Asn-Ala linker via RF-cloning. His_6_-smt3-G2_TEV_-Ub was created by inserting the full Tobacco Etch Virus protease (TEV) cleavage site C-terminal to the mG2 via a modified RF-cloning protocol.

### Protein expression and purification

All constructs were expressed in *Escherichia coli* BL21(DE3) cells in Luria broth [42], and grown at 37°C until an optical density at 600 nm (OD_600_) of 0.6 was reached. Cells were induced by addition of IPTG were left overnight at 16°C. For His_6_-CISD1, FeCl_3_ (800 µM) was added to the media to facilitate the expression of the Fe^3+^-bound protein. All human parkin (full-length, R0RBR, ^77^R0RBR) and Miro1 (mG2, mG2-Ub, mG2_TEV_-Ub) constructs were encoded as His_6_-Smt3 fusion proteins. For purification, cells were harvested, resuspended in lysis buffer (50 mM Tris, 500 mM NaCl, 0.5 mM triscarboxyethylphosphine (TCEP), 25 mM imidazole, pH 8.0) and lysed using an EmulsiFlex-C5 homogenizer (Avestin). All parkin constructs were purified by Ni^2+^-NTA affinity chromatography by HisTrap FF column on an AKTA FPLC (Cytiva Life Sciences, Vancouver, Canada), eluted with 500 mM imidazole, and the affinity tag was cleaved by Ulp1 protease (1 : 50 protease:protein). After cleavage, the proteins were passed again through the HisTrap FF column and the flowthrough was collected, concentrated and buffer exchanged by gel filtration column (Superdex75 10/300 GL column, Cytiva Life Sciences). Finally, the purified parkin was aliquoted and flash frozen for storage at −80°C. For all Miro1 constructs, the proteins were purified using Ni^2+^-NTA Sepharose. After batch binding for 1 h at 4°C, proteins were eluted and rapidly diluted before overnight incubation with Ulp1 protease in 50 mM Tris, 200 mM NaCl, 100 mM Arginine Hydrochloride (ArgHCl), 250 µM TCEP, pH 8.0. The cleaved proteins were then passed through the Ni^2+^-NTA Sepharose and the flowthrough was collected, concentrated and buffer exchanged by gel filtration. The final purified protein was aliquoted and stored at −80°C.

Yeast Ub and human CISD1 (residues 32–108) were encoded as His_6_- fusion proteins, containing a TEV protease site for affinity tag cleavage. His_6_-Ub, His_6_-CISD1 and His_6_-CISD1-Ub proteins were loaded onto a Ni^2+^-NTA Sepharose column, as above, and eluted using 400 mM imidazole. The His_6_- tags were cleaved by adding TEV (1 : 50 protease:protein) and cleaved overnight at 4°C. Proteins were repassed over the Ni^2+^-NTA Sepharose, the flowthrough collected, and further purified using gel filtration. Miro1^181–579^ was purified as previously described by Kumar et al. [11]. *P. humanus* PINK1 was encoded as a glutathione S-transferase (GST-) fusion, purified as previously described [24]. All proteins were aliquoted and flash frozen at −80°C.

### Preparation of phosphorylated parkin, ubiquitin and acceptor-Ub proteins

Purified GST-PINK1 was incubated with full length parkin (1 : 2), ubiquitin (1 : 10) or substrate-Ub (1 : 20) proteins and dialyzed against buffer containing 50 mM Tris, 100 mM NaCl, 1 mM DTT, 100 mM ArgHCl (mG2-Ub only), 10 mM MgCl_2_ and 10 mM ATP (pH 7.0) at 25°C for 16 h. Protein phosphorylation was monitored to completion using Phos-Tag AAL^TM^ (NARD Institute Ltd) SDS–PAGE. Following the reaction, GST-PINK1 was removed using a GSTrap FF column, collecting the flowthrough fractions containing the phosphorylated proteins and buffer exchanged by gel filtration. The phospho-proteins were buffer exchanged using a Superdex75 10/300 GL column (Cytiva Life Sciences).

### Preparation of mG2^K572^-Ub

Equimolar amounts of mG2 and His_6_-Ub (180 µM) were incubated at 37°C with Uba1 (50 nM), UBE2L3 (4 µM), pParkin (75 nM) and ATP (10 mM) for up to 2 h. To isolate mG2^K572^-Ub, the reaction was purified by Ni^2+^-NTA affinity chromatography and TEV protease cleavage, as described above. The final mG2^K572^-Ub was flash frozen and stored at −80°C.

### Preparation of fluorescently labelled proteins

Ubiquitin, pUb, mG2, mG2-Ub, mG2_TEV_-Ub, mG2^K572^-Ub, CISD1 or CISD1-Ub each with an added N-terminal cysteine residue were expressed and purified as described above. Purified Ub was modified with DyLight 800 maleimide (Thermo Fisher, Waltham, MA, U.S.A.) at a 1 : 1.5 ratio for 1 h at pH 7.4 and quenched with 5 mM DTT. All other proteins above were modified with a 1 : 0.8 ratio protein:Alexa Fluor 680 (Thermo Fisher, Waltham, MA, U.S.A.) for 10 min at pH 7.4 and quenched with 5 µl β-mercaptoethanol. Parkin was modified with a 1 : 0.8 ratio of Dylight 800 and quenched with 5 µl β-mercaptoethanol. Excess DyLight 800 or Alexa Fluor 680 label was removed using a HiLoad Superdex75 gel filtration column (Cytiva Life Sciences). Aliquots of labelled protein were flash frozen and stored at −80°C until needed.

### Protein interaction experiments

Isothermal titration calorimetry (ITC) experiments were performed using a NanoITC (TA Instruments, New Castle, DE, U.S.A.) at either 18°C or 25°C. All experiments were done in duplicate using freshly prepared proteins in 25 mM HEPES or 20 mM Tris, 100 mM NaCl and 250 µM TCEP at pH 7.5 or pH 8.0. Parkin (20 µM in 162 µl) was titrated with mG2-Ub (100 µM), CISD1-Ub (212 µM), mG2-pUb (84 µM) or CISD1-pUb (106 µM) using 2 µl injection volumes and 300 s intervals between injections. Data were analysed using single-site binding models using NanoAnalyze software (TA Instruments).

All microscale thermophoresis (MST) experiments were performed using a Monolith NT.115 (NanoTemper, Munich, Germany). Measurements were performed at 25°C using 60% LED and 40% MST power, with laser off/on times of 5 and 30 s, respectively. His_6_-Smt3-Parkin constructs were labelled with Monolith His-Tag Labelling Kit RED-tris-NTA 2nd Generation (Cat#MO-L018) according to the supplied labelling protocol. A titration series of up to 16 dilutions was generated from 1 : 1 serial dilutions starting from 50 µM unlabelled Miro1^181–579^ in PBS-T buffer (PBS 1×, 0.05% Tween 20). Binding affinities were determined by using a 1 : 1 binding interaction, and data were fitted with a Hill equation (Hill coefficient = 1):

$$h(x)=\frac{C_{max}}{1+\frac{K_{d}}{x}}$$

where *C_max_* is the fluorescence difference between the bound and unbound state, and *K*_d_ is the dissociation constant. The fit was performed using least-square minimization method in GNUPLOT software.

Sedimentation velocity experiments were conducted using a Beckman XL-A analytical ultracentrifuge equipped with an An60Ti rotor. Double sector cells (1.2 cm) with quartz windows were filled with 380 µl sample and 400 µl reference buffer. All data were collected in 50 mM Tris, 200 mM NaCl, 0.5 mM TCEP pH 8.0 at 18°C (mG2-Ub) or 20 mM Tris, 100 mM NaCl, 1 mM DTT pH 8.0 at 20°C (CISD1-Ub). All experiments were performed at 45 000 rpm. Cells were scanned at equal intervals, 45 scans total, and sedimentation was monitored by absorbance at 250 nm (mG2-Ub) or 280 nm (CISD1-Ub). Data were processed by c(s) distribution analysis in SEDFIT v15.01, accounting for equilibration and rotor acceleration times. For parkin, mG2-Ub and CISD1-Ub, partial specific volumes ($\bar{\nu }$) were 0.719 ml g^−1^, 0.739 ml g^−1^, 0.7377 ml g^−1^, respectively, viscosity (η) was 0.01033 Poise, and density (ρ) was 1.0079 g/ml. Sedimentation coefficients observed in buffer were corrected for 20°C in water (S_20,w_), and frictional coefficients (f/f_0_) were calculated from S_20,w_ and known molecular weights of all species. All data were fit to an RMSD < 0.005 in SEDFIT.

### Ubiquitination assays

Most reactions were completed with 4 µM Ub, 1 µM ^800^Ub, 0-2 µM pUb, 1 µM (p)Parkin, 2 µM substrate (mG2, mG2-Ub), 0.5 µM UBE2L3, 5 mM MgATP, 50 mM HEPES, pH 7.5. For CISD1-(p)Ub reactions, the reactions contained 1 µM UBE2L3, 1 µM parkin, 1 µM CISD1-Ub, 1 µM pUb and 20 µM Ub. All assays were initiated by adding 0.1 µM Uba1, reacted at 37°C and quenched at desired time points using 3× SDS sample buffer and 1 µl of 1 M DTT. Gradient gels (4–12% Bis-Tris Plus, Thermo Fisher Scientific) were used with MES running buffer (50 mM MES, 50 mM Tris, 0.1% SDS, 1 mM EDTA, pH 7.3). Gels were scanned by Odyssey Imaging system (LiCor) and fluorescence intensity was measured at 700 nm and 800 nm.

Western blots of assays were processed on a PVDF membrane using a semidry iBlot2 apparatus (Invitrogen). Membranes were rinsed with TBS and blocked with 10% BSA (Thermo Fisher, Waltham, MA, U.S.A.) in TBS 10% aqueous Tween 20 (TBST) for 1 h at room temperature. A final rinse with TBST was used prior to incubation with primary antibody (ɑ-parkin, Bio-Techne, catalog #AF1438) overnight at 4°C. Following this, membranes were rinsed with three to four washes of TBST, incubated with a fluorescent secondary antibody (anti-Goat Alexa 680 nm, Invitrogen, catalog #A-21088) for 45 min, and washed again three times with TBST. Membranes were scanned by an Odyssey Imaging system (LiCor), and fluorescence intensity was measured at 700 nm.

### mG2_TEV_-Ub cleavage ubiquitination assays

mG2_TEV_-pUb reactions contained 0.5 μM UBE2L3, 1 µM (p)Parkin, 5 µM Ub, 2 µM mG2t_TEV_-pUb and were initiated by 0.1 µM Uba1. After 15 min, the reactions were quenched by adding 2 µl apyrase (1 mg/ml) and 1 µM EDTA. For cleavage, 2 µl of TEV protease (2.5 mg/ml) was added and the reactions were incubated for an additional 60 min at 37°C. Uncleaved reactions had 2 µl of water added. All samples were quenched with 3× SDS sample buffer and run on 4–12% Bis-Tris gels in MES running buffer. All proteins were visualized by Coomassie staining.

## Results

### Neither pParkin nor pParkin:pUb are sufficient for substrate ubiquitination

**Figure 1.**
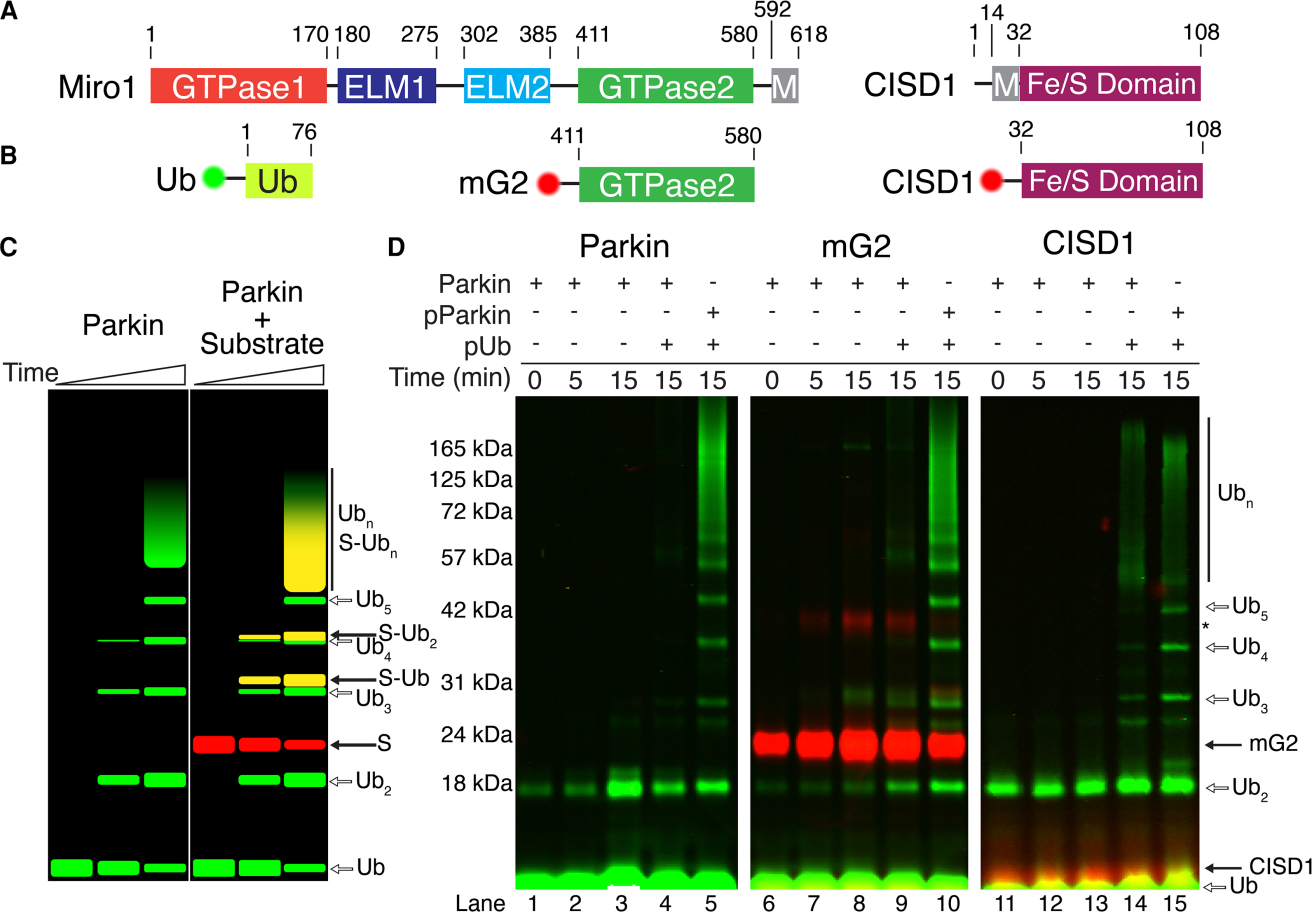
Parkin shows minimal ubiquitination activity with an isolated substrate. (**A**) Schematic diagram showing the domains of human Miro1 and human CISD1. (**B**) Schematic diagram showing the minimal target constructs mG2 and CISD1 used in this study. Also indicated are the N-terminal fluorescent labels for mG2, CISD1 (Alexa Fluor 680, red circle) and Ub (DyLight 800, green circle). (**C**) Model assay demonstrating the expected outcomes of single-fluorescent autoubiquitination (left) and dual-fluorescent substrate (right) assays. (**D**) Parkin autoubiquitination and minimal substrate ubiquitination were monitored by fluorescently labelled ^800^Ub (green) and ^680^mG2 or ^680^CISD1 (red). Reactions were initiated by addition of Uba1 (E1) and quenched at the required timepoints with 3× sample buffer and DTT.

Parkin autoubiquitination and free ubiquitin (Ub) chain formation are frequently monitored as proxies for parkin ligase activity. In the absence of a substrate, parkin ubiquitination is activated through pUb binding and enhanced following PINK1 phosphorylation of the parkin Ubl domain. Since *in vivo* ubiquitination experiments reflect a combination of translocation and ubiquitination effects both with distinct temporal dependencies, we sought to develop an *in vitro* method where we could control each step of parkin activation while monitoring autoubiquitination, free Ub chain formation and target protein ubiquitination simultaneously. This removes the dependency on translocation to the OMM while providing tight control over the stoichiometry of phosphorylation and allows us to distinguish the relative importance of the two PINK1 phosphorylation events on the different types of ubiquitination. In all experiments, parkin does not contain an N-terminal affinity- or immuno-tag, known to alter the activity of the E3 ligase [7,29] and result in extensive auto-ubiquitination of the tag itself [23,43]. We also included two well-documented parkin target proteins: Miro1 and CISD1 (Figure 1A), which are both located on the OMM and have been shown in multiple studies to be ubiquitinated by parkin under oxidative stress conditions [11,15,22,27,29,35,38,44]. Miro1 is a multi-domain GTPase anchored to the OMM at its C-terminus. Previous studies have indicated that K572 in the GTPase2 domain of Miro1 is the primary site of parkin ubiquitination [5,15,27,29] so we created a protein construct, mG2, that contains only the C-terminal GTPase2 domain of human Miro1 (residues 411–580) (Figure 1B). Likewise, the redox protein CISD1 (MitoNEET) lacking only its transmembrane domain (Figure 1B) (residues 32–108) was created. Thus, both proteins contained all identified parkin-mediated ubiquitination sites [5,15,29]. To distinguish among the different possible Ub products that can be generated in an *in vitro* assay, proteins were fluorescently tagged with Alexa Fluor 680 (Miro1 and CISD1) or with a DyLight 800 fluorophore (Ub) (Figure 1B). This strategy allowed products formed on Miro1 or CISD1 (red fluorescence) to be distinguished from all other products (green fluorescence) in a single experiment using simultaneous fluorescence detection (Figure 1C).

In the absence of either Miro1 or CISD1, typical *in vitro* ubiquitination assays show that robust formation of Ub products requires the presence of pUb and PINK1-phosphorylated parkin (pParkin) (Figure 1D, lanes 1–5). Consistent with many other studies [11,12,31], the products were confirmed to be parkin-mediated free Ub chains rather than poly-ubiquitinated parkin, as Western blot analyses of the assays show a single band for parkin rather than ubiquitin laddering (Supplementary Fig. S1). Upon addition of either target protein, free-Ub chain synthesis by parkin remains efficient, with minimal ubiquitination of mG2 or CISD1 under identical conditions (Figure 1D, lanes 6–15, noted by the lack of red fluorescent bands). These data show that PINK1-mediated phosphorylation of parkin and Ub results in a preference for free Ub chain synthesis with little modification of isolated OMM substrate proteins such as Miro1 and CISD1.

### Phospho-ubiquitin is an anchor for acceptor/target proteins with parkin

For any post-translational modification, an enzyme and substrate are required to interact, even minimally, for a successful reaction. Thus parkin and OMM proteins such as Miro1 and CISD1 require a physical interaction to facilitate ubiquitin transfer. The data from Figure 1 suggests this interaction is very weak or insufficient to stimulate or orient the substrate for ubiquitination. To assess this, we used three different methods to measure the direct interactions between parkin and either Miro1 or CISD1. We were unable to detect a direct interaction between parkin and either, a near full-length, soluble construct of Miro1 that lacks only its GTPase1 domain (residues 181–579, Miro1^181–579^) or the entire extramembranous portion of CISD1 (Supplementary Fig. S2), using either microscale thermophoresis (MST) or isothermal titration calorimetry (ITC). These observations support the lack of ubiquitination of Miro1 or CISD1 from our *in vitro* experiments (Figure 1). Based on the concentrations used in these experiments these data indicate that the interactions of CISD1 or Miro1^181–579^ with parkin are extremely weak with estimated dissociation constants (*K*_d_) greater than 100 µM. As this value is well above the expected cellular concentrations, we hypothesized that OMM proteins such as Miro1 and CISD1 do not interact directly with parkin and require an accessory factor to facilitate ubiquitination by parkin.

**Figure 2.**
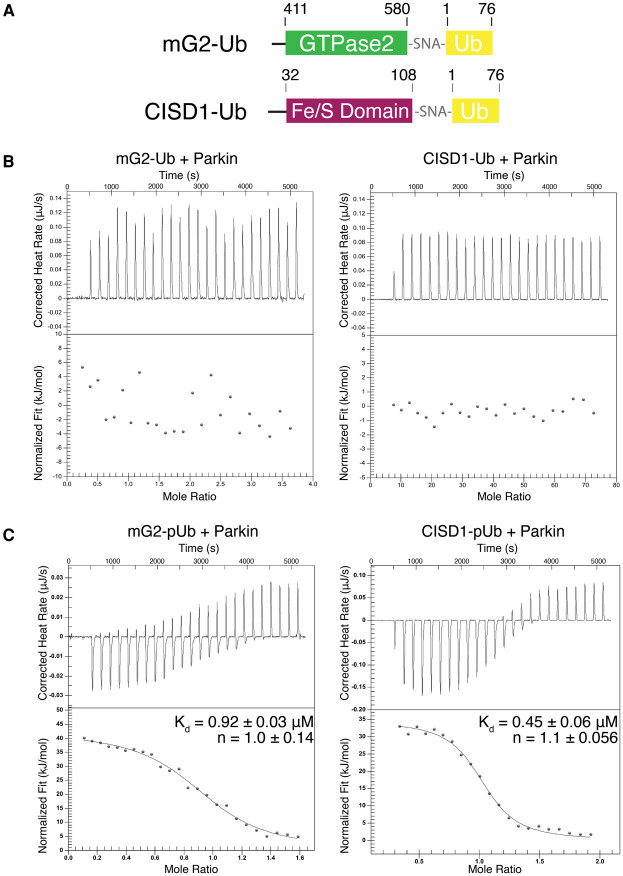
Parkin interacts with a substrate-pUb. (**A**) Schematics of chimeric constructs mG2-Ub and CISD1-Ub. (**B** and **C**) Isothermal titration calorimetry of parkin (20 µM) titrated with mG2-Ub (100 µM), CISD1-Ub (212 µM), mG2-pUb (84 µM) or CISD1-pUb (106 µM). The *K*_d_ values shown are an average of duplicate titrations. mG2-pUb titrated into parkin had a calculated ΔH = 41 ± 5.5 kJ/mol and ΔS = 260 ± 19 J/mol K. CISD1-pUb titrated into parkin had a calculated ΔH = 30 ± 6.3 kJ/mol and ΔS = 220 ± 22 J/mol K.

It has been proposed that pre-ubiquitination of an OMM protein by an endogenous mitochondrial E3 ligase [23] and subsequent phosphorylation of that Ub by PINK1 accelerates parkin recruitment to damaged mitochondria [14] and enhances ubiquitination of artificial substrates such as maltose-binding protein [43]. To identify how each of these events might control parkin-mediated recruitment of OMM proteins, we created two chimeric proteins where the N-terminus of Ub was fused to the C-termini of mG2 and CISD1, to serve as proxies of pseudo-ubiquitinated target proteins (mG2-Ub and CISD1-Ub, Figure 2A). We measured the strengths of interaction for each of these chimeric proteins with parkin using ITC (Figure 2B) and analytical sedimentation velocity experiments (Supplementary Fig. S3A). As with our results using near full-length Miro1 and CISD1 alone we could not observe a measurable interaction between parkin and either mG2-Ub or CISD1-Ub by either method. This result indicates that Ub attached to either mG2 or CISD1 does not promote an association with parkin. These results are consistent with *in vivo* experiments that show parkin is not translocated to the OMM in the absence of PINK1 phosphorylation of Ub [14,34,37,45].

It has been shown that parkin translocation to the OMM is dependent on phosphorylation of pre-existing K48- and K63-linked chains that have subsequently been phosphorylated [14,15]. To determine how phosphorylation of an OMM protein might alter its interaction with parkin, we used PINK1 to phosphorylate each chimera at S65 of its Ub (mG2-pUb or CISD1-pUb) and purified the resulting products. In contrast with the unphosphorylated chimeras, tight binding is observed between mG2-pUb or CISD1-pUb and parkin when measured by ITC (Figure 2C) or MST (Supplementary Fig. S2A) or analytical ultracentrifugation (Supplementary Fig. S3B) with measured dissociation constants (*K*_d_) of 0.92 ± 0.03 µM and 0.45 ± 0.06 µM (ITC), respectively. In ITC experiments the endothermic heat change and dissociation constants are similar to the parameters measured for the isolated pUb interaction with parkin [11,12,37] suggesting that there are no additional interactions between the target mG2 or CISD1 proteins and parkin that would be expected to increase the observed affinities. Our results indicate that phospho-ubiquitin is the primary determinant by which an interaction between an OMM protein and parkin occurs. This is consistent with *in vivo* experiments that show PINK1 phosphorylation of ubiquitin is the most important element for parkin translocation to the OMM [14,34,37,45].

**Figure 3.**
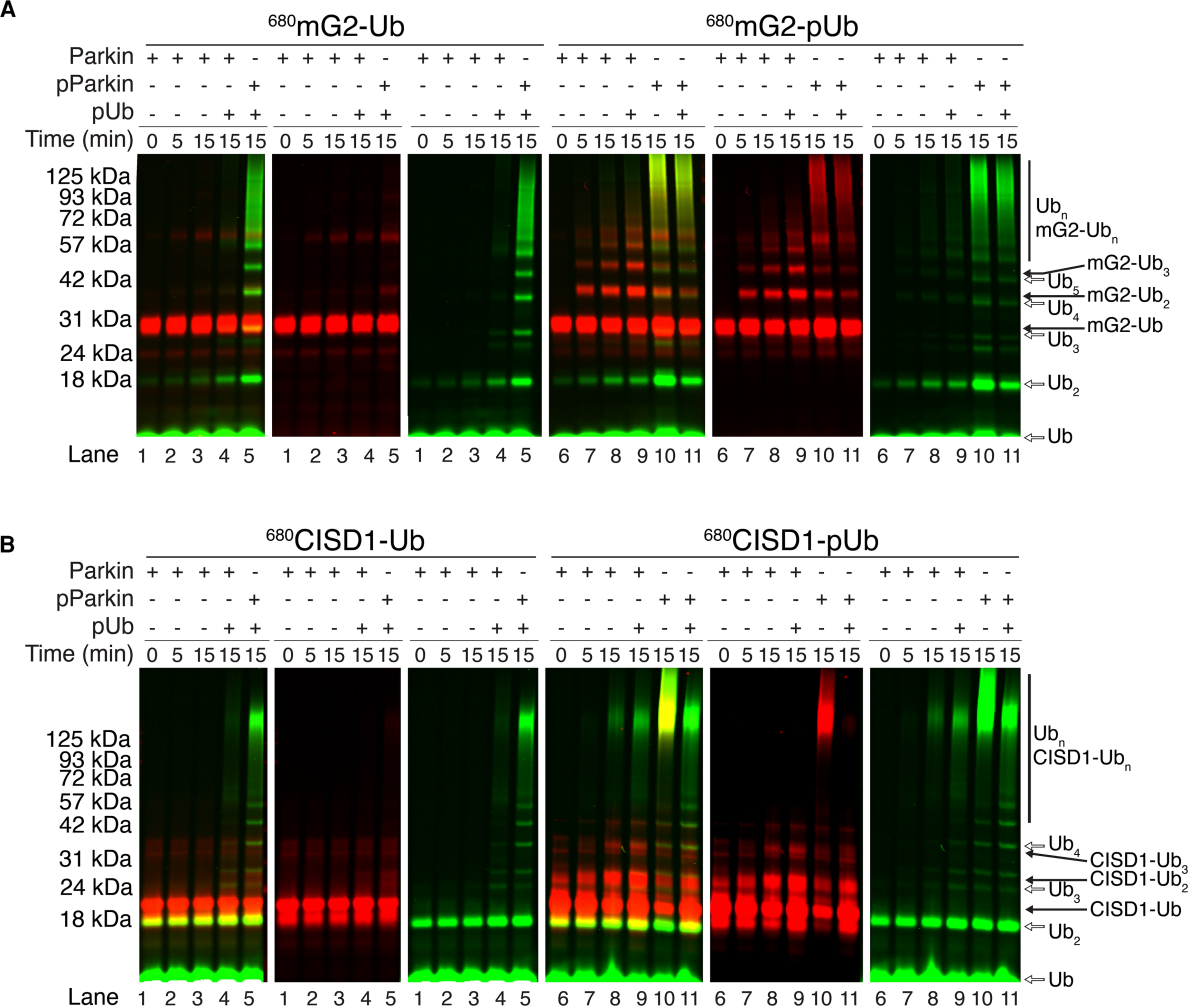
Parkin poly-ubiquitinates the acceptor-pUb. Parkin autoubiquitination of (**A**) mG2-(p)Ub or (**B**) CISD1-(p)Ub ubiquitination were monitored by fluorescently labelled ^800^Ub (green) and ^680^mG2-Ub, ^680^mG2-pUb, ^680^CISD1-Ub and ^680^CISD1-pUb (red). Reactions were initiated by addition of Uba1 (E1) and quenched at the required timepoints with 3× sample buffer and DTT. For each experiment three panels are shown filtered for both green and red (left), red only (centre) and green only (right) fluorescence.

We hypothesized that the pUb module of the mG2-pUb or CISD1-pUb species serves to anchor and position the attached protein for efficient ubiquitination by parkin. Since no recognition motif for parkin ubiquitination is evident in known mitochondrial substrates and our data show no direct substrate interactions with parkin, we introduce the term ‘acceptor’ to refer to the mitochondrial protein, as typical substrates have direct contact with an enzyme. We conducted parkin-mediated ubiquitination assays using the mG2-Ub and CISD1-Ub chimeras. In the presence of pUb and phosphorylated parkin, assays with mG2-Ub (Figure 3A, lanes 1–5) or CISD1-Ub (Figure 3B, lanes 1–5) show a high degree of free Ub chain formation (green fluorescence) very similar to that noted in the absence of an OMM protein (Figure 1). Little ubiquitination of either mG2 or CISD1 occurs even for pParkin in the presence of pUb, as shown by the lack of red fluorescent bands on the gel (Figure 3, second panel, lanes 4, 5 for each). This observation is consistent with the poor complex formation between parkin and either mG2-Ub or CISD1-Ub shown in Figure 2B (also Supplementary Figs. S2, S3A). In contrast, phosphorylation of mG2-Ub (mG2-pUb) leads to robust ubiquitination of the chimeric protein by parkin, likely due to enhanced proximity driven by the affinity of the pUb moiety, as evidenced by the red fluorescence spaced at regular molecular weight intervals (Figure 3, lanes 6–9). Though not as striking, efficient ubiquitination of CISD1-pUb is evident based on the decrease in intensity of the unmodified CISD1-pUb band (Figure 3B, lane 8). We note particularly that in the presence of mG2-pUb and unphosphorylated parkin, ubiquitination of the mG2-pUb species with little free Ub chain formation is observed (Figure 3A, lane 8). Upon phosphorylation of the Ubl domain in parkin, and in the presence of a pUb chimera the overall ubiquitination activity increases, although a marked increase in free Ub chains is evident that is not present with unphosphorylated parkin (Figure 3, lanes 10–11). These results suggest that phosphorylation of Ub on an OMM protein is essential for recruitment of parkin and sufficient to promote further ubiquitination of the OMM accepter protein. Phosphorylation of parkin appears to improve this but also induces a by-product of free Ub chain formation *in vitro* (Figure 3A, red and green lanes 10–11).

As verification that the high molecular weight ubiquitin species are not parkin autoubiquitination products when in the presence of mG2-pUb, the reactions were repeated to directly monitor parkin ubiquitination by fluorescent labelling (^800^Parkin) and immunoblotting (^680^α-Parkin) (Supplementary Fig. S4). Both detection methods confirmed that parkin does not extensively ubiquitinate itself, even when phosphorylated. Therefore, the high molecular weight species are either multi-ubiquitinated acceptor-pUb species and/or free Ub chains.

### Parkin phosphorylation state influences acceptor ubiquitination versus free ubiquitin chains

**Figure 4.**
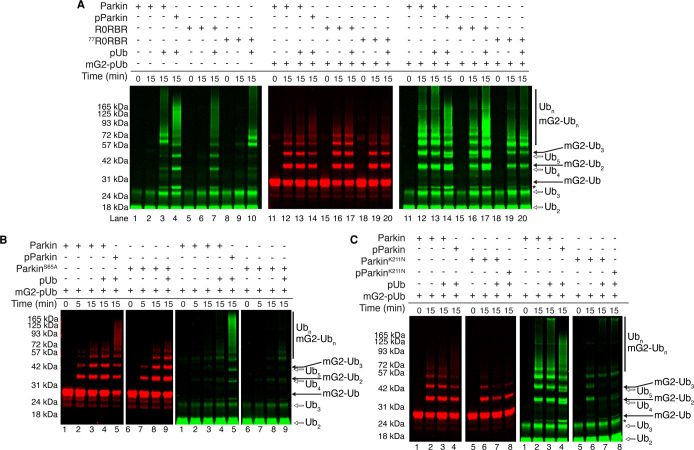
The Ubl domain is not required for mG2-pUb poly-ubiquitination. Ubiquitination assay using fluorescently labelled ^800^Ub (green) and ^680^mG2-pUb (red) to test the activity of (**A**) parkin and two truncated parkin species, R0RBR and ^77^R0RBR. The left panel shows ubiquitination in the absence of mG2-pUb whereas the centre and right panels show ubiquitination experiments filtered for ubiquitination of mG2-pUb (red) and overall ubiquitination (green), respectively. Ubiquitination of mG2-pUb (red) and overall ubiquitination (green) using (**B**) parkin^S65A^ and (**C**) parkin^K211N^. Reactions were initiated by addition of Uba1 (E1) and quenched at the required timepoints with 3× sample buffer and DTT.

Our acceptor-pUb chimeras show that anchoring an OMM protein to parkin via a linked pUb moiety facilitates ubiquitination while limiting free Ub chain formation. The ligase activity towards an acceptor-pUb chimera in the absence of PINK1-mediated phosphorylation of parkin implies this step is not essential for initial acceptor ubiquitination by parkin. These observations may indicate that phosphorylation of the parkin Ubl domain acts as a switch for directed ubiquitination of the acceptor versus uncontrolled ubiquitination. To test this hypothesis, we created two truncated parkin proteins, lacking either the Ubl-linker domains (R0RBR, residues 141–465) or only the Ubl domain (^77^R0RBR, residues 77–465) to assess Ubl-independent acceptor ubiquitination. R0RBR parkin retains its ability to recruit mG2-pUb but does not interact with Miro1^181–579^ (Supplementary Fig. S2). In the absence of an acceptor both R0RBR and ^77^R0RBR proteins had lower free Ub chain-building activity compared with full-length parkin, even in the presence of pUb (Figure 4A, compare lanes 5–10 with lanes 1–4). Remarkably, introducing mG2-pUb leads to significant ubiquitination increases of mG2-pUb by both R0RBR and ^77^R0RBR. Specifically, the patterns of ubiquitination (red fluorescence) for R0RBR and ^77^R0RBR (Figure 4A, lanes 16–17 and 19–20) match very closely to that observed using unphosphorylated parkin (Figure 4A, lanes 12–13). These results clearly show that the phosphorylated Ubl domain is not necessary to direct parkin ubiquitination towards an OMM protein. To confirm this finding in the context of full-length parkin, we tested parkin constructs containing an S65A substitution in the Ubl domain (parkin^S65A^) or K211N (parkin^K211N^) in the RING0 domain. As shown in multiple *in vivo* studies, parkin^S65A^ poorly synthesizes free Ub chains in the presence of pUb [24,25,27,32]. However, in the presence of an acceptor with phosphorylated ubiquitin (mG2-pUb), parkin^S65A^ displays robust ubiquitination of mG2-pUb (Figure 4B, lanes 6–9) that is nearly identical with that observed for wild-type parkin with the absence of free Ub chains. (Figure 4B, lanes 1–4). Similarly, parkin^K211N^ loses its ability to form free Ub chains upon phosphorylation of the Ubl domain or in the presence of free pUb (Figure 4C, green, lanes 5–8). In contrast, upon addition of the mG2-pUb acceptor, the results were markedly different. For both parkin^K211N^ and pParkin^K211N^, we observed ubiquitination, albeit diminished, of the mG2-pUb species (Figure 4C, red) with the near absence of free Ub chain formation. These results show that the phosphorylated Ubl domain is not an absolute requirement for acceptor ubiquitination but does have a significant influence on the generation of free Ub chains. This latter finding agrees with marked decreases in autoubiquitination and free Ub chain formation observed based on structural models that show the Ubl domain, phosphorylated at S65, has the potential to interact with a basic region of the RING0 domain (K161, K163, K211) [25,26].

The ubiquitination of the chimeric acceptors requires the pUb anchor as an essential component since chimeras where parkin's N-terminal Ubl domain was tethered to mG2 in place of Ub did not drive ubiquitination onto the acceptor, even upon phosphorylation (Supplementary Fig. S5). The mG2-pUbl chimera failed to activate either acceptor- or free Ub chain formation with all parkin variants, including those lacking the Ubl domain (Supplementary Fig. S5). These results indicate that OMM target protein ubiquitination by parkin is specific for the attached pUb anchor and phosphorylation of parkin contributes to activity in a different manner.

To rule out the possibility that the C-terminal -GG sequence in the Ub moieties of each chimeric protein could be used to promote ubiquitin chain formation, rather than free Ub, we synthesized fluorescently labelled mG2 that was mono-ubiquitinated at K572 (mG2^K572^-Ub), phosphorylated it using PINK1 and conducted similar ubiquitination assays (Supplementary Fig. S6). Under these conditions where the conjugated C-terminus of Ub is blocked, we observed robust multi-ubiquitination of mG2^K572^-pUb (Supplementary Fig. S6, red, lanes 7–8) by unphosphorylated parkin, with minimal improvement upon parkin phosphorylation. In contrast, the unphosphorylated conjugate mG2^K572^-Ub showed evidence for free Ub chain formation with only minimal ubiquitination of the acceptor under all conditions (Supplementary Fig. S6, red, lanes 4–5). Together, these results indicate that a pUb module linked to an acceptor is the essential requirement for acceptor ubiquitination by parkin.

### Acceptors are ubiquitinated, not their phospho-ubiquitin anchors

**Figure 5.**
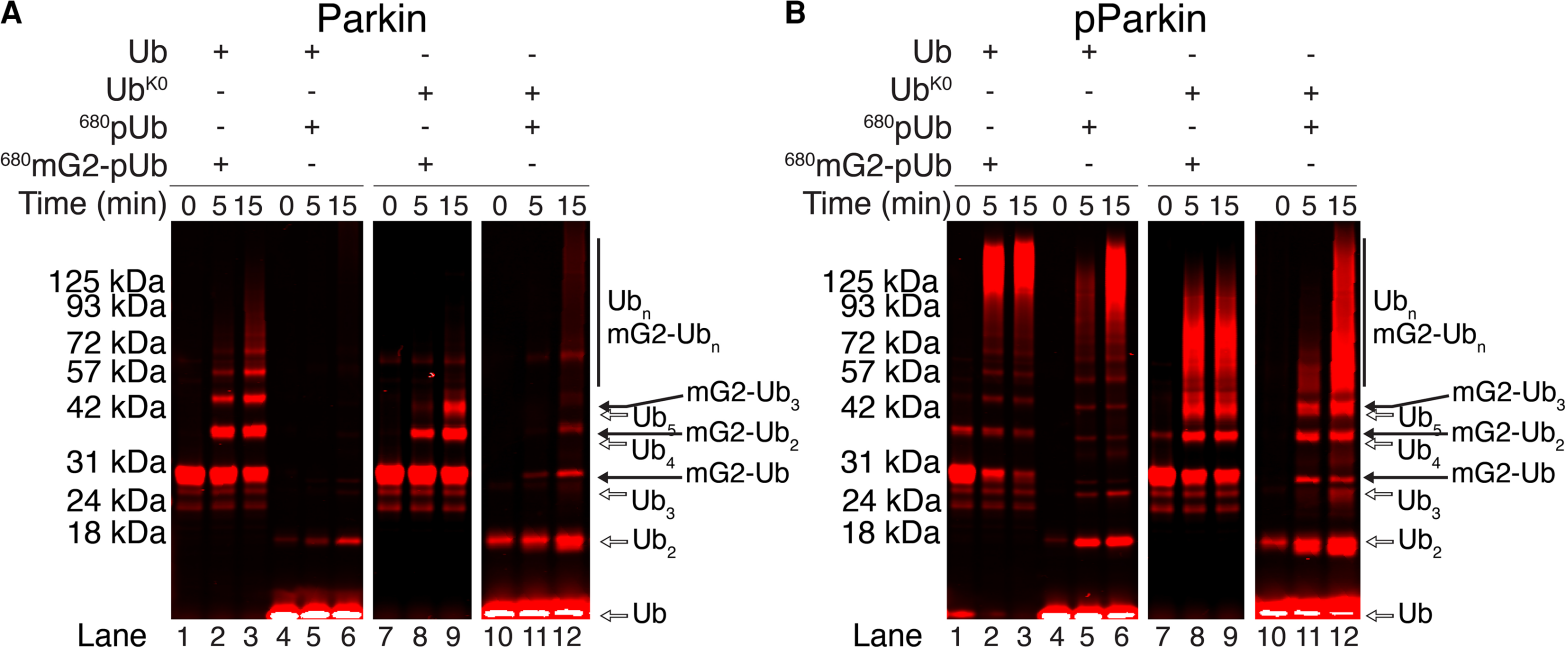
Unphosphorylated parkin fails to ubiquitinate pUb. Ubiquitination assays using fluorescently labelled ^680^mG2-pUb (red) and ^680^pUb (red) to compare the method of ubiquitination by parkin (**A**) and pParkin (**B**). Reactions utilized either wild-type Ub or Ub with all lysine residues substituted to arginine (Ub^K0^) and were initiated by addition of Uba1 (E1) and quenched at the required timepoints with 3× sample buffer and DTT. Only red fluorescence arising from ubiquitination of ^680^mG2-pUb (lanes 1–3, 7–9) or ^680^pUb (lanes 4–6, 10–12) is visible.

In our system we have shown that phosphorylated Ub linked to the OMM proteins Miro1 and CISD1 stimulates parkin ubiquitination of the chimeric proteins. It is possible that either the mG2/CISD1 acceptor or pUb anchor components in the chimeras are ubiquitinated in our assays, leading to potentially different biological outcomes. For example, ubiquitination of the OMM proteins would provide a signal for mitophagy, while ubiquitination of the pUb protein may be an indirect result of the chimeric protein and have little relationship to mitochondrial removal. We first measured the ability of unphosphorylated parkin to ubiquitinate mG2-pUb compared with free pUb, using either fluorescently labelled ^680^mG2-pUb as an indicator of ubiquitination on the acceptor mG2 protein or ^680^pUb as an indicator of free chain formation on the pUb moiety (Figure 5). In these assays, we observed extensive ubiquitination on mG2-pUb using unphosphorylated parkin (Figure 5A, lane 3) similar to our previous results (Figs. 3, 4). In contrast, when ^680^pUb lacking the mG2 protein was used, only minimal di-ubiquitin formation was observed with little evidence of higher molecular weight free Ub chains (Figure 5A, lane 6). When the same assays were carried out with pParkin, we observed very different results. In the presence of ^680^mG2pUb we observed robust ubiquitination by pParkin (Figure 5B, lanes 2–3) although the pattern of ^680^mG2-pUb bands on the gel were different from those using unphosphorylated parkin. When only fluorescent ^680^pUb was used, an intense ubiquitination pattern was evident, as for ^680^mG2-Ub, judged by the red fluorescence (Figure 5B, lanes 5–6). This indicates ubiquitin labelling must be occurring on the pUb moiety when using pParkin. The simplest interpretation of this data is that unphosphorylated parkin targets the acceptor mG2 protein. While phosphorylation of parkin amplifies this signal, ubiquitination is mostly directed towards the pUb moiety rather than the acceptor protein, resulting in a loss of target specificity.

We repeated these reactions using lysine-free Ub (Ub^K0^) to determine whether parkin is building poly-ubiquitin chains or mono-ubiquitinating multiple lysines on ^680^mG2-pUb or ^680^pUb. This data shows that mG2-pUb is efficiently modified with at least two ubiquitins, suggesting that parkin utilizes at least two lysine residues on the mG2-pUb before extending any chains (Figure 5A, lane 9). Using ^680^pUb alone with parkin the presence of Ub^K0^ resulted in a minor amount of di-ubiquitin being formed (Figure 5A, lane 12), again showing the preference for unphosphorylated parkin to ubiquitinate the acceptor mG2 protein. Similar experiments using phosphorylated parkin (pParkin) lead to reduced ubiquitin laddering with ^680^mG2-pUb. In the presence of ^680^pUb only, a number of distinct band representing Ub_2_, Ub_3_ and Ub_4_ are evident (Figure 5B, lanes 7–12). These data support the idea that unphosphorylated parkin directs ubiquitin ligation to a discrete number of lysines on a phospho-ubiquitinated acceptor protein rather than forming extensive poly-ubiquitin chains. On the other hand, phosphorylation of parkin appears to switch ubiquitination sites away from the acceptor protein resulting in ubiquitination of the pUb protein.

**Figure 6.**
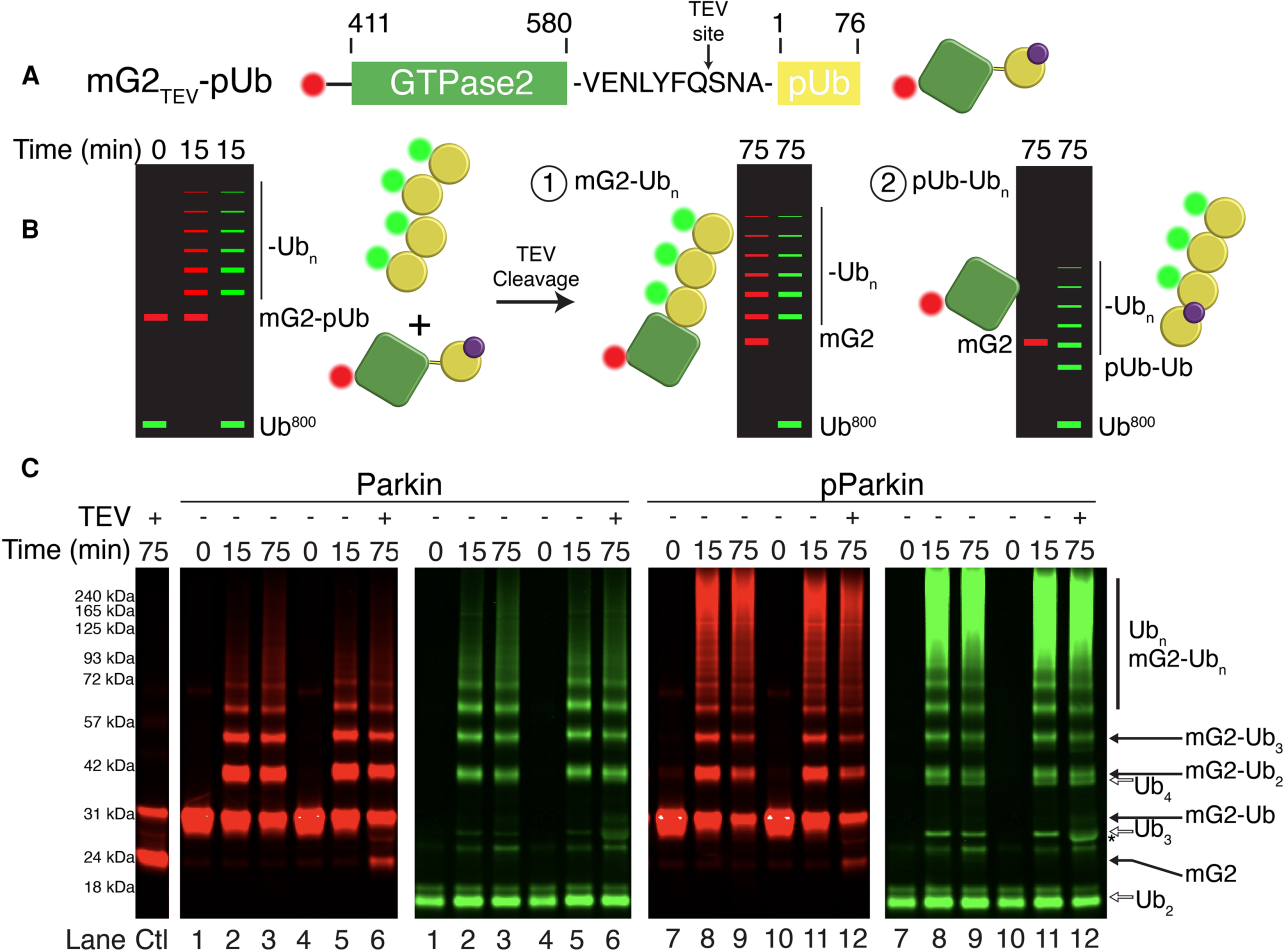
Parkin ubiquitinates the substrate and not the pUb. (**A**) Schematic diagram of the TEV cleavable mG2_TEV_-Ub chimera. (**B**) Model diagrams and results demonstrating the possible results after ubiquitination and cleavage of mG2_TEV_-pUb. (**C**) Ubiquitination assays using fluorescently labelled ^800^Ub (green) and ^680^mG2_TEV_-pUb (red). The duplicate parkin (left) and pParkin (right) reactions were left for 15 min before quenching with apyrase and EDTA to stop parkin activity. TEV protease was added to cleave the ubiquitinated substrate for an additional 60 min. Control lane (Ctl) shows parallel cleavage of unmodified mG2_TEV_-pUb over the allowed time.

To further identify whether the OMM component of an acceptor-pUb is ubiquitinated, we inserted a tobacco etch virus protease (TEV) cleavage site (VENLYFQ^SN) between the fluorescently labelled mG2 and pUb moieties that would allow proteolytic cleavage of the chimeric protein, mG2_TEV_-Ub (Figure 6A). Following ubiquitination and subsequent TEV cleavage, two outcomes are possible with respect to the red fluorescent mG2 protein (Figure 6B). In the first scenario, ubiquitination on the mG2 would yield a red-fluorescent multi-ubiquitin ladder that has been shifted on a gel by the mass of the single phospho-ubiquitin tag that has been cleaved. Alternatively, if ubiquitination occurs on the pUb moiety, a collapse of the red fluorescent ladder to a single mG2 band and shifting of the green ubiquitin ladder to a lower position on the gel by the mG2 mass should be observed. Consistent with our previous results, ubiquitination of mG2_TEV_-Ub by parkin yielded discrete bands, a result of ubiquitination of the mG2-pUb chimera (Figure 6C, lanes 1–3). This pattern, along with free Ub chains is evident in reactions using phosphorylated parkin (pParkin, Figure 6C, lanes 7–9). These reactions were repeated (Figure 6C lanes 4–5, 10–11) followed by cleavage of mG2_TEV_-Ub using TEV protease (Figure 6C, lanes 6, 12). Although we achieved incomplete TEV cleavage of the chimera (∼75%, Figure 6C, Ctl lane), the data for unphosphorylated parkin clearly show both the appearance of mG2 (lower red band, lane 6) and the retention of ubiquitin products on mG2 (upper red bands, lane 6). In addition, there was little evidence of green Ub_3_ and Ub_4_ bands expected if ^800^Ub labelled the pUb moiety (green, lane 6). In contrast, TEV cleavage of mG2 from pParkin ubiquitination reactions yield a weaker mG2 band (lower red band, lane 12), bands for ubiquitinated mG2 species (red upper bands, lane 12) and had clear bands for Ub_3_, and Ub_4_ species (green, lane 12), indicative of ubiquitination of the pUb protein. Together with data from Figure 5, these data indicate that parkin-mediated ubiquitination of mG2_TEV_-pUb is directed towards the acceptor mG2 protein, using the attached pUb to anchor it to parkin. Upon phosphorylation of parkin, further ubiquitination is re-directed to include ubiquitination of the pUb itself resulting in free poly-ubiquitin chains.

## Discussion

**Figure 7.**
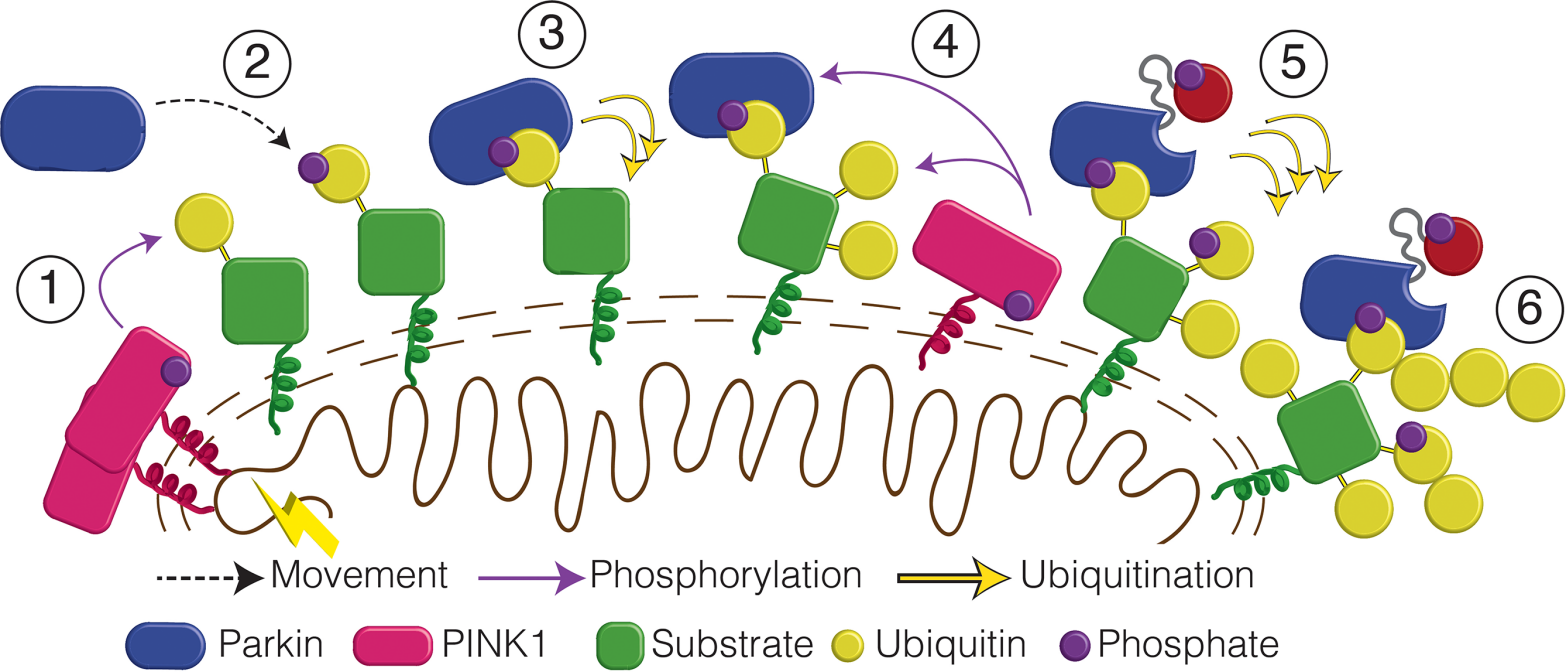
Schematic of divergent parkin ubiquitination activity. (**1**) PINK1 phosphorylates nearby Ub molecules in existing K48- and K63-linked chains in outer mitochondrial membrane proteins. (**2**) Parkin is recruited to mitochondria by surface pUb molecules. (**3**) Parkin multi-ubiquitinates the attached mitochondrial proteins. (**4**) New Ub molecules are phosphorylated, thus amplifying the signal. (**5**) Parkin is eventually phosphorylated by PINK1 which increases parkin's ubiquitination activity, primarily through free Ub chain formation. (**6**) Unrestricted ubiquitination creates the poly-ubiquitin signal required to stimulate mitophagy.

Understanding the mechanisms of activation and substrate ubiquitination by the E3 ligase parkin are key to identifying its role in early-onset PD and the development of potential therapeutics. A key first step involves parkin translocation to damaged mitochondria in response to oxidative stress. This is dependent on the presence of PINK1-phosphorylated pUb [22,23], assembled primarily in K48- and K63-linked chains by several constitutive mitochondrial E3 ligases [23,46,47] (Figure 7). *In vivo* experiments that show translocation and ubiquitination of OMM proteins by parkin are negligible in the absence of pUb at the OMM. Once localized to the OMM, parkin ubiquitinates surface exposed proteins as an initial signal for mitophagy. This tight coupling between translocation and parkin activity makes it difficult to dissect ubiquitination efficiencies due to temporal differences in translocation rates observed in cells. For example, experiments using ΔUbl-parkin (^77^R0RBR) show that translocation to the OMM is negligible compared with wild-type parkin after 60 min [37], resulting in poor ubiquitination. Yet other studies show that despite poor translocation to the OMM, parkin^S65A^ retains the ability to ubiquitinate some OMM proteins [22,34,37]. To uncouple these events, we designed chimeric OMM proteins (Miro1, CISD1) linked to a single Ub protein that could be phosphorylated by PINK1 and used in ubiquitination activity reactions. We recognize that a possible disadvantage of this approach is that linking Ub to the C-terminus of either Miro1 or CISD1 may not adequately represent the normal process of lysine ubiquitination. This is offset by several advantages including the capability to achieve precise stoichiometry of an OMM protein interaction with parkin, the decoupling of the phosphorylation of Ub from that of parkin and the ability to fluorescently label all components of the ubiquitination reaction to identify substrate ubiquitination, free Ub chain formation and autoubiquitination processes.

Our work shows that direct interactions of Miro1 or CISD1 with parkin are very weak, unobservable by several methods, even when these proteins are ubiquitinated. This translates into poor ubiquitination of these OMM proteins. Notably, the overall ubiquitination activity of phosphorylated parkin in the presence of phospho-ubiquitin was significant in the absence or presence of an OMM protein. Yet, the majority of this activity was directed at free Ub chain formation, rather than ubiquitination of the OMM protein itself. This result has been noted by many other groups, from both *in vivo* and *in vitro* experiments, even when parkin is phosphorylated [11,12,16,22,27,30–32].

Phosphorylation of ubiquitin within in our chimeric proteins results in tight interactions with parkin (*K*_d_ < 1 µM) that are similar to that for pUb alone. This results in robust ubiquitination of the acceptor protein, which is especially evident in the absence of parkin phosphorylation (Figure 7). In our chimeric Miro1 construct (mG2-pUb) we demonstrate that unphosphorylated parkin directs ubiquitin to the Miro1 acceptor protein rather than the pUb moiety. In contrast, phosphorylation of the parkin Ubl domain increases the overall ubiquitination activity, as previously observed [15,16,22,25,26,28] but switches the ubiquitination profile towards free Ub chain formation as opposed to OMM acceptor ubiquitination. As a result, parkin proteins lacking the entire N-terminus (R0RBR), the Ubl domain (^77^R0RBR) or substituted to prevent phosphorylation (parkin^S65A^) exhibit significant ubiquitination of chimeric mG2-pUb with minimal formation of free Ub chains. These observations establish that pUb, rather than the OMM protein itself, acts to guide and orient the acceptor protein near parkin to facilitate ubiquitination. It is possible that these observations are specific for the two proteins studied in this work (Miro1, CISD1). However, our observations are supported from early studies that show binding of pUb to parkin greatly stimulates Ub discharge from an E2∼Ub conjugate transfer [27] and improves ubiquitination activity, even in the absence of phosphorylation of parkin [14,15,23]. Structural studies show this likely arises due to pUb binding to the RING0-RING1 cleft in parkin that re-organizes the IBR domain away from the Ubl domain [11,12,25,26,30,36] and allows recruitment of a UBE2L3∼Ub conjugate [30,48]. Furthermore, previous studies show that unphosphorylated parkin efficiently ubiquitinates a green-fluorescent protein tagged with pUb [23] or an N-terminal-tag tethered to parkin [7,43]. Our results provide a rationale for the broad spectrum of proteins ubiquitinated by parkin under oxidative stress conditions [5,49]. More than 30 OMM proteins, including Miro1/2, CISD1, mitofusins 1/2 and proteins in the TOM complex have been identified that bear little sequence similarity suggesting a common parkin recognition motif. Furthermore, there is little information to suggest direct interaction of these proteins with parkin, a requirement for ubiquitination. Immunoprecipitation assays from cell lysates have hinted at interactions between parkin and some substrates including Miro1 [35,38] and mitofusin 2 [39] that are enhanced after oxidative stress, perhaps due to pUb tagging of each of these proteins.

Recent crystal structures indicate that the increased ubiquitination activity of parkin may rely on the ability of the S65-phosphorylated Ubl domain to relocate from its RING1 binding site to a basic patch comprising K161 and K211 in the RING0 domain [25,26]. Our experiments show that parkin lacking its Ubl domain (R0RBR), parkin^S65A^ and the early-onset PD substitution K211N retain the ability to ubiquitinate mG2-pUb but appear to have decreased formation of free Ub chains. These are consistent with observations by multiple other groups [25–27,29,31,37] that show decreased free Ub chain formation or autoubiquitination. One possibility is that pUbl relocation may stimulate the formation of free Ub chains or autoubiquitination rather than substrate ubiquitination. Curiously, the PD substitutions K161N and K211N also lead to reduced translocation of parkin to the OMM upon oxidative stress [19,21,37]. This observation seems inconsistent with a role in parkin recruitment as these residues are remote from the RING0-RING1 pUb binding site. One possibility is that K161/K211 acts as a secondary pUb binding site in addition to the well-characterized RING0-RING1 cleft that assists in translocation. Such an idea would rationalize the ubiquitination of chimeric-pUb proteins observed here in the absence of parkin phosphorylation. This would be especially important for OMM proteins carrying ubiquitin chains with multiple phosphorylated Ub molecules that would have the potential to coordinate multiple pUb binding sites in parkin. Indeed, indirect evidence for this second site exists from *in vitro* biophysical experiments where tighter pUb binding is observed in K161N- or K211N-substituted parkin [14] compared with wild-type parkin and direct pUb titration experiments of pUb-bound R0RBR that show evidence of weak, additional pUb binding [17].

Our observations indicate that acceptor-pUb species are multi-ubiquitinated by parkin, in the absence of phosphorylation of its Ubl domain and that subsequent phosphorylation of parkin results in enhanced free Ub chain formation and minor parkin autoubiquitination. This divergent mechanism accelerates ubiquitination of mitochondrial proteins upon initial recruitment of parkin to the OMM, without relying on phosphorylation of the Ubl domain (Figure 7). Instead, this mechanistic step ensures a greater number of Ub molecules are available for PINK1 phosphorylation and subsequent parkin recruitment, contributing to a feed-forward mechanism of parkin mediated ubiquitination [22,23]. Over 70 substitutions in parkin have been associated with early-onset PD and their impact on parkin function remains unpredictable with current data [21,37]. The observations from this work will be important for influencing further structural and functional studies for elucidating parkin's mechanism of substrate ubiquitination and potential therapeutic intervention. Our study offers important insight into the effects of parkin activation and substrate choice, and thus has implications for the rational design of therapeutics for mitochondrial targeting.

## Data Availability

All data are contained within the manuscript. All reagents and protein constructs will be made available by the authors upon request.

## Abbreviations

ArgHCl: Arginine Hydrochloride
GST-: glutathione S-transferase
IBR: in-Between-RING
ITC: isothermal titration calorimetry
OMM: outer mitochondrial membrane
PD: Parkinson's disease
PINK1: PTEN-induced kinase 1
RF: Restriction-Free
TCEP: triscarboxyethylphosphine
TEV: Tobacco Etch Virus protease
