# Supplementary material for: Distinct phosphorylation signals drive acceptor versus free ubiquitin chain targeting by parkin
Source: Biochem J. 2022 Mar 28;479(6):751–66. doi: 10.1042/BCJ20210741 (PMC9022993; doi:10.1042/BCJ20210741)
Supplement: Supplementary Material [file BCJ-479-751-s1.pdf]

Supplementary Figure Legends

Figure S1: Parkin phosphorylation promotes free Ub chain formation. Parkin autoubiquitination assays using fluorescently labelled ^{800}Ub. Ubiquitin was visualized by in-gel fluorescence at 800 nm (left) and parkin was detected by Western Blot and a fluorescently labelled secondary antibody (Alexa 680) (right).

Figure S2: **Parkin does not interact with Miro1 or CISD1. (**A) MST experiment titrating 16 dilutions of Miro1$^{181\text{-}579}$ (black triangle) or mG2-pUb (red circles) into His_6-Smt3-tagged parkin (left) and R0RBR (right). (**B**) Isothermal titration calorimetry of parkin (20 µM) titrated with CISD1 (200 µM).

Figure S3: **Parkin interacts with mG2-pUb and CISD1-pUb.** For mitochondrial acceptors mG2 and CISD1, sedimentation velocity experiments were conducted to determine the interaction with parkin. In **(A)**, sedimentation of parkin alone (grey), mG2-Ub or CISD1-Ub alone (dashed) or a mixture of parkin with either mG2-Ub or CISD1-Ub (black) shows the sedimentation coefficient does not change for parkin indicating the proteins do not interact at the concentrations used. In **(B)** the same experiments are completed using either mG2-pUb or CISD1-pUb alone (dashed) that show a clear shift in sedimentation coefficient for parkin (black) indicative of an interaction. The two signals in **(B)** for CISD1-Ub are due to both protomer and dimer forms of the protein.

Figure S4: **Parkin phosphorylation, even in the presence of mG2-pUb, promotes free Ub chain formation.** Parkin autoubiquitination assays using various fluorescent labels to detect ubiquitination activity. ^{680}mG2-pUb, ^{800}Ub and ^{800}parkin were visualized by in-gel fluorescence and parkin was also detected by Western Blot and a fluorescently labelled secondary antibody (Alexa 680) (right).

Figure S5: **A linked acceptor-pUbl cannot induce ubiquitination by parkin or ΔUbl parkin.** In (A) and (B) ubiquitination using the parkin Ubl domain fused to the C-terminus of mG2 in the absence of phosphorylation (mG2-Ubl) or after phosphorylation using PINK1 (mG2-pUbl) was tested. In (A) assays were conducted using parkin or pParkin in the presence and absence of pUb. In (B) assays were conducted using two ΔUbl parkin constructs, R0RBR and ^{77}R0RBR in the presence and absence of pUb. Reactions were initiated by addition of Uba1 (E1) and quenched at the required timepoints with 3x sample buffer and DTT.

Figure S6: **Parkin efficiently multi-ubiquitinates a truly mono-ubiquitinated substrate, mG2^{K572}-Ub.** (A) Schematic diagrams and cartoon representations comparing mG2-pUb to mG2^{K572}-pUb (B) Preparative ubiquitination reaction (lanes 1-3), purification (lanes 4-9) and fluorescent labeling (lanes 10-21) of mono-ubiquitinated mG2^{K572}-Ub and mG2K572-pUb. (C) Parkin auto-ubiquitination and mG2^{K572}-Ub ubiquitination were monitored by fluorescently labelled 800Ub (green) and ^{680}mG2^{K572}-Ub or ^{680}mG2^{K572}-pUb (red). Reactions were initiated by addition of Uba1 (E1) and quenched at the required timepoints with 3x sample buffer and DTT. The mG2-Ub_2 observed at t = 0 min was a by-product of preparative ubiquitination of mG2 and could not be separated during purification.

Figure S1

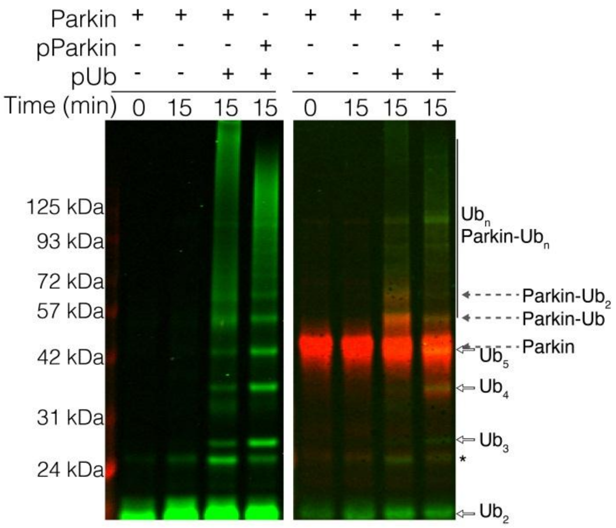

Parkin + + + - + + + -
pParkin - - - + - - - +
pUb - - + + - - + +
Time (min) 0 15 15 15 0 15 15 15
125 kDa
93 kDa
72 kDa
57 kDa
42 kDa
31 kDa
24 kDa
Ub$_n$
Parkin-Ub$_n$
Parkin-Ub$_2$
Parkin-Ub
Parkin
Ub$_5$
Ub$_4$
Ub$_3$
*
Ub$_2$

Figure S2

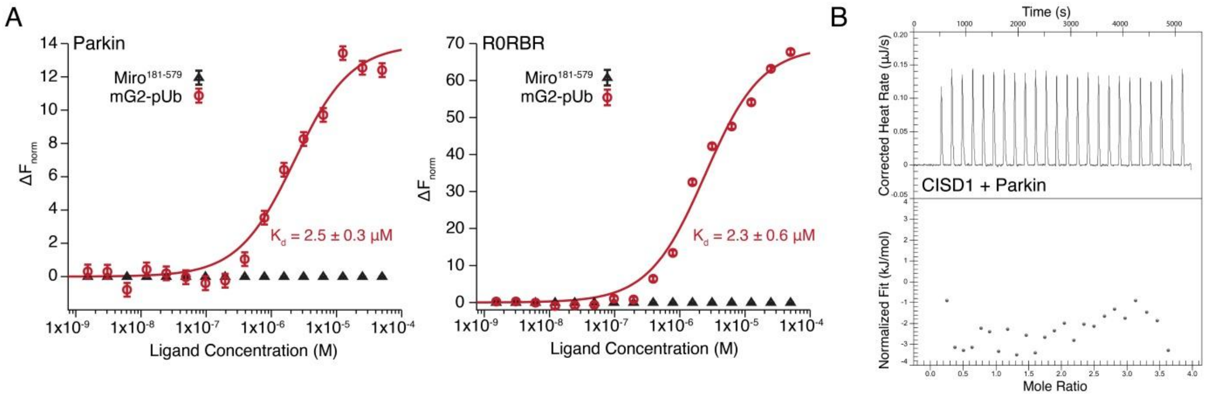

A
Parkin
Miro181-579
mG2-pUb
ΔFnorm
Kd = 2.5 ± 0.3 μM
Ligand Concentration (M)
R0RBR
Miro181-579
mG2-pUb
ΔFnorm
Kd = 2.3 ± 0.6 μM
Ligand Concentration (M)
B
Time (s)
Corrected Heat Rate (μJ/s)
CISD1 + Parkin
Normalized Fit (kJ/mol)
Mole Ratio

Figure S3

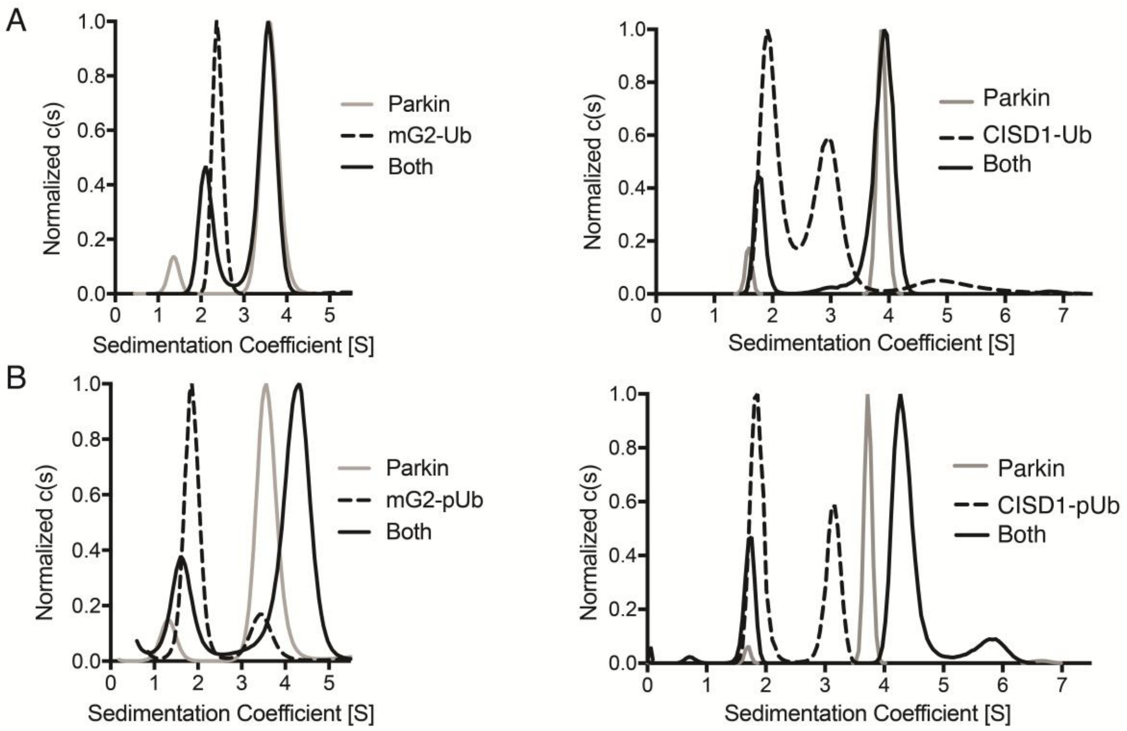

A
B
Normalized c(s)
Sedimentation Coefficient [S]
Parkin
mG2-Ub
Both
CISD1-Ub
mG2-pUb
CISD1-pUb

Figure S4

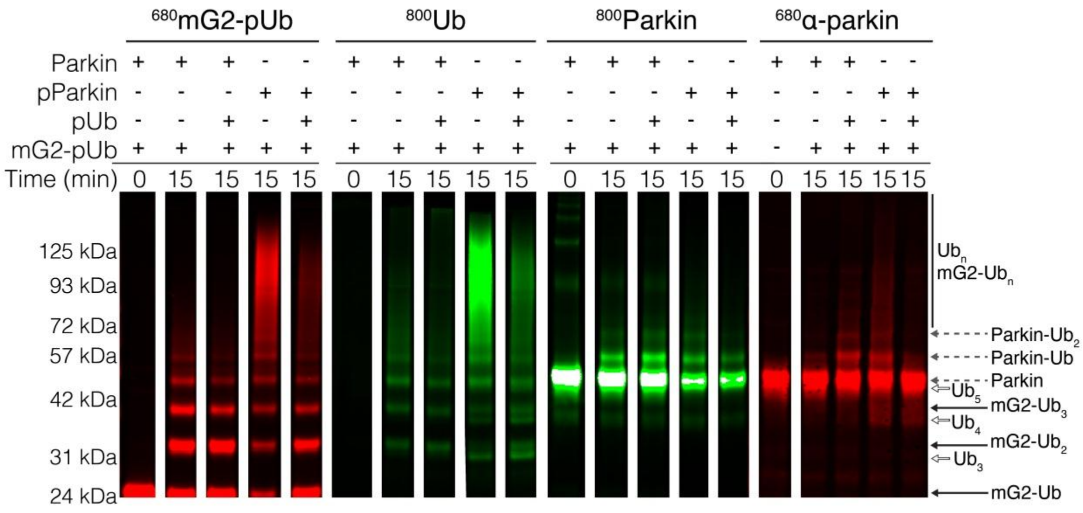

680mG2-pUb
800Ub
800Parkin
680α-parkin
Parkin
pParkin
pUb
mG2-pUb
Time (min)
125 kDa
93 kDa
72 kDa
57 kDa
42 kDa
31 kDa
24 kDa
Ubn
mG2-Ubn
Parkin-Ub2
Parkin-Ub
Parkin
Ub5
mG2-Ub3
Ub4
mG2-Ub2
Ub3
mG2-Ub

Figure S5

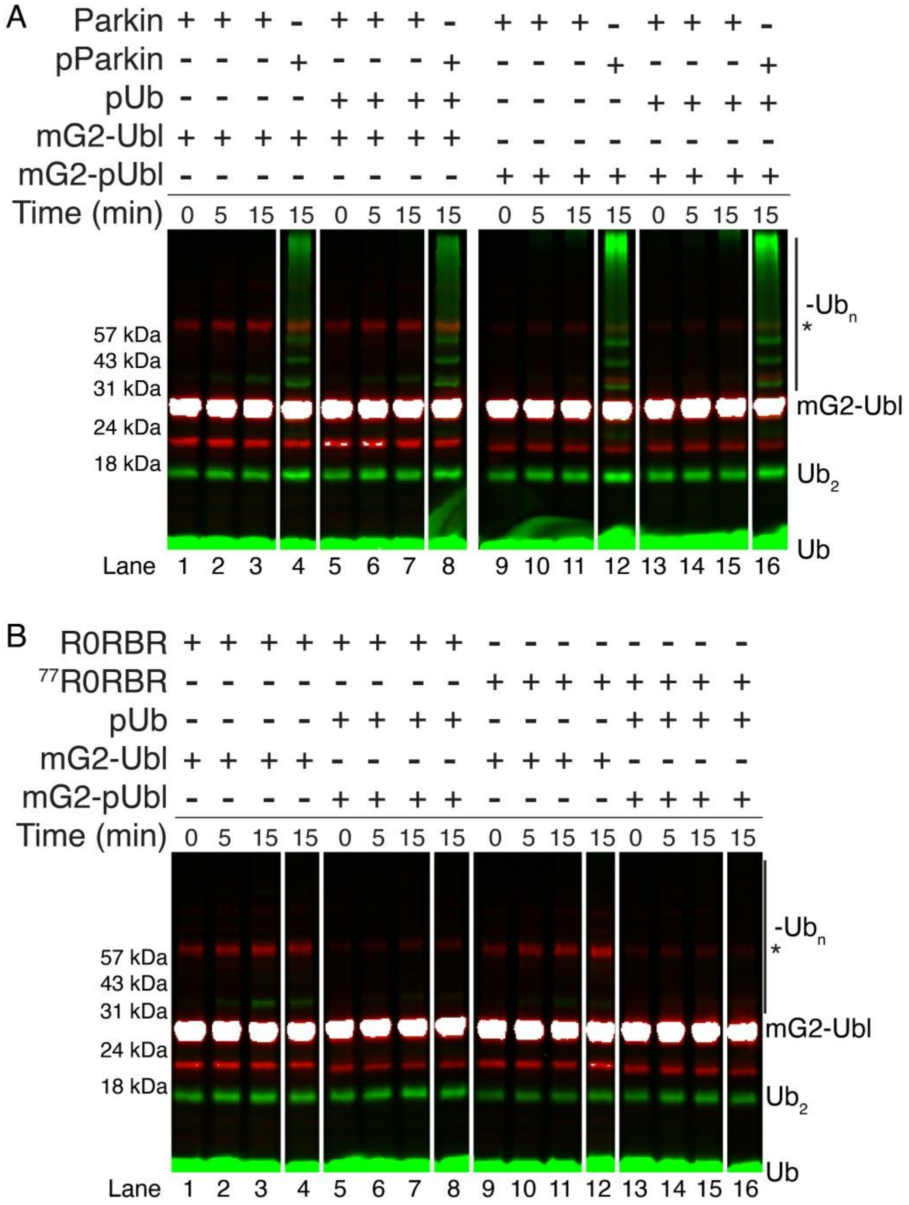
A
Parkin
pParkin
pUb
mG2-Ubl
mG2-pUbl
Time (min)
0 5 15 15 0 5 15 15 0 5 15 15 0 5 15 15
57 kDa
43 kDa
31 kDa
24 kDa
18 kDa
-Ubn
*
mG2-Ubl
Ub2
Ub
Lane 1 2 3 4 5 6 7 8 9 10 11 12 13 14 15 16
B
R0RBR
77R0RBR
pUb
mG2-Ubl
mG2-pUbl
Time (min)
0 5 15 15 0 5 15 15 0 5 15 15 0 5 15 15
57 kDa
43 kDa
31 kDa
24 kDa
18 kDa
-Ubn
*
mG2-Ubl
Ub2
Ub
Lane 1 2 3 4 5 6 7 8 9 10 11 12 13 14 15 16

Figure S6

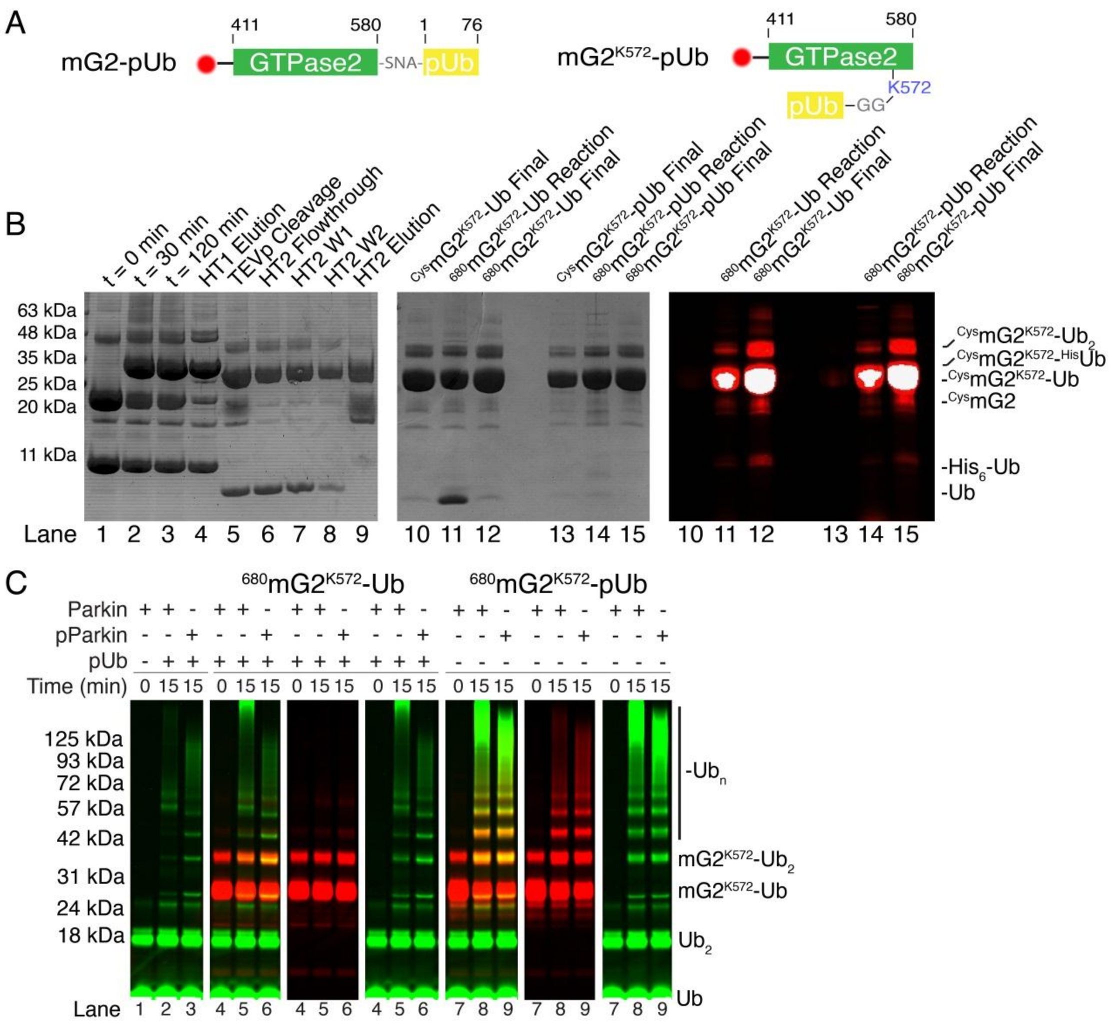
A
mG2-pUb
411
580
1
76
GTPase2
-SNA-
pUb
mG2K572-pUb
K572
GG
B
t = 0 min
t = 30 min
t = 120 min
HT1 Elution
TEVp Cleavage
HT2 Flowthrough
HT2 W1
HT2 W2
HT2 Elution
CysmG2K572-Ub Final
680mG2K572-Ub Reaction
680mG2K572-Ub Final
CysmG2K572-pUb Final
680mG2K572-pUb Reaction
680mG2K572-pUb Final
63 kDa
48 kDa
35 kDa
25 kDa
20 kDa
11 kDa
CysmG2K572-Ub2
CysmG2K572-HisUb
CysmG2K572-Ub
CysmG2
His6-Ub
Ub
Lane 1 2 3 4 5 6 7 8 9 10 11 12 13 14 15 10 11 12 13 14 15
C
680mG2K572-Ub
680mG2K572-pUb
Parkin
pParkin
pUb
Time (min)
125 kDa
93 kDa
72 kDa
57 kDa
42 kDa
31 kDa
24 kDa
18 kDa
Ubn
mG2K572-Ub2
mG2K572-Ub
Ub2
Ub
Lane
